# Liver transplantation in a patient with hepatitis B, C and D coinfection associated with hepatocellular carcinoma: a management strategy for a rare condition. Case report: Transplante hepático em paciente com coinfecção pelos vírus B, C e D associada a carcinoma hepatocelular: uma estratégia de tratamento para uma condição rara. Relato de caso

**DOI:** 10.1590/1516-3180.2015.8881501

**Published:** 2015-07-03

**Authors:** Lucas Carvalho Dantas, Tércio Genzini, Marcelo Perosa de Miranda, Regina Gomes dos Santos, Nilton Ghiotti de Siqueira, Judith Weirich, Cirley Maria de Oliveira Lobato

**Affiliations:** I MD. Physician, Health Sciences Center, Universidade Federal do Acre (UFAC), Rio Branco, Acre, Brazil; II MSc. Physician, Director and Founder, Liver Transplantation Group, Hospital Beneficência Portuguesa, São Paulo, São Paulo, Brazil; III MD. Physician, Liver Transplantation Group, Hospital Beneficência Portuguesa, São Paulo, São Paulo, Brazil.; IV MD, PhD. Physician, General Surgery Unit, Hospital das Clínicas do Acre, Rio Branco, Acre, Brazil; V MD, MSc. Physician, Hepatology and Tropical Diseases Unit, Hospital das Clínicas do Acre, Rio Branco, Acre, Brazil; VI MD, PhD. Physician, Hepatology and Tropical Diseases Unit, Hospital das Clínicas do Acre, Rio Branco, Acre, Brazil

**Keywords:** Hepatitis B, Hepatitis C, Hepatitis D, Carcinoma, hepatocellular, Liver transplantation

## Abstract

**CONTEXT:**

Orthotopic liver transplantation (OLT) is the treatment of choice for end-stage liver disease. Cirrhosis due to hepatitis C infection is the leading indication for liver transplantation worldwide. However, patients who are given transplants because of viral liver diseases often present clinical coinfections, including hepatitis B together with hepatitis D. Currently, different strategies exist for patient management before and after liver transplantation, and these are based on different protocols developed by the specialized transplantation centers.

**CASE REPORT:**

We present a rare case of a 58-year-old man with chronic hepatitis B, C and D coinfection. The patient developed cirrhosis and hepatocellular carcinoma. His treatment comprised antiviral therapy for the three viruses and OLT. The patient's outcome was satisfactory.

**CONCLUSION:**

OLT, in association with antiviral therapy using entecavir, which was administered before and after transplantation, was effective for sustained clearance of the hepatitis B and D viruses. A recurrence of hepatitis C infection after transplantation responded successfully to standard treatment comprising peginterferon alfa-2A and ribavirin.

## Abstract (pt)

**CONTEXTO:**

O transplante ortotópico de fígado (TOF) é o tratamento de escolha em pacientes com doença hepática terminal. A cirrose por hepatite C é a principal indicação de transplante hepático no mundo. No entanto, pacientes transplantados por hepatopatias virais frequentemente apresentam coinfecções, como hepatite B associada a hepatite D. Atualmente, existem diferentes estratégias de manejo em pacientes pré e pós-transplantados conforme diferentes protocolos de conduta de serviços especializados em transplante.

**RELATO DE CASO:**

Apresentamos o raro caso de um homem de 58 anos diagnosticado com as hepatites crônicas B, C e D. O paciente evoluiu com cirrose e carcinoma hepatocelular. O tratamento consistiu de terapia antiviral para os três vírus e de transplante ortotópico de fígado. O desfecho do paciente foi satisfatório.

**CONCLUSÃO:**

O transplante ortotópico de fígado, associado à terapia antiviral com entecavir antes e após o procedimento, foi eficaz na depuração sustentada dos vírus B e D. A recidiva do vírus C após o transplante respondeu com sucesso ao tratamento padrão com alfapeginterferon 2A e ribavirina.

## INTRODUCTION

Orthotopic liver transplantation (OLT) is the treatment of choice for end-stage liver disease. Hepatitis C virus (HCV)-associated cirrhosis is the most common indication for OLT worldwide.^1^^,^ 2 However, patients who are given transplants secondary to viral liver diseases often present with coinfections, including infections with the hepatitis B virus (HBV) and hepatitis D virus (HDV).^3^ Different strategies exist for patient management before and after OLT, and these are based on protocols developed by the different transplantation centers.

Here, we report a rare case of a patient coinfected with HBV, HCV and HDV in association with hepatocellular carcinoma (HCC). The treatment strategy led to a satisfactory outcome. 

## CASE REPORT

A 58-year-old non-alcoholic man was experiencing short episodes of upper gastrointestinal bleeding and was referred to the Hepatology and Tropical Diseases Unit of Acre, in the Amazon region of northern Brazil, in October 2008. 

Clinical examination revealed a distended abdomen and hepatosplenomegaly. An ultrasound scan showed impairment of the hepatic parenchyma, a hyperechoic nodule in the right lobe and signs of portal hypertension with severe ascites.

### Diagnosis

The serological and virological tests were consistent with a diagnosis of chronic hepatitis caused by HBV and HDV coinfection. A real-time polymerase chain reaction (RT-PCR) for HBV deoxyribonucleic acid (TaqMan; Roche Diagnostics AG, Rotkreuz, Switzerland) showed high viral loads (log 6.22), and HDV ribonucleic acid (RNA) was detected using the same method. Serological tests for HCV were negative, and serum alanine transaminase (ALT) levels were five times higher than the normal upper limit. The patient began antiviral treatment with entecavir (1.0 gram once a day).

A pretreatment liver biopsy showed impairment of the hepatic parenchyma, moderate periportal inflammation and piecemeal necrosis with severe focal cell damage and cirrhosis (Metavir score: A3; F4). 

A computed tomography scan of the liver nodules showed three hypervascular lesions in the right lobe. The largest of these was in segment VI, and it had a diameter of 5.0 centimeters. The two other nodules were in segments VII and VIII, and both had diameters of 1.9 centimeters. These findings led to a preliminary diagnosis of suspected HCC of viral etiology caused by HBV and HDV coinfection, which is an indication for liver transplantation based on the modified Milan criteria used in Brazil.^4^^-^ 6

The patient's model for end-stage liver disease (MELD) score was 14, and he was put on the liver transplantation waiting list. Despite the MELD score of 14, the patient's good clinical condition led the doctors to opt for segmental hepatectomy of the right lobe involving segments V and VI, which extended to resection of the largest nodule. This procedure was followed by observation of the patient with an expectation of further liver resections or an OLT.^6^

Histopathological analysis on the liver confirmed the presence of a well-differentiated multifocal HCC without vascular invasion and without lymph node involvement.

The patient's condition deteriorated rapidly and progressively during the days after the procedure, and he experienced gastrointestinal bleeding caused by a variceal hemorrhage. The variceal hemorrhage was controlled using an endoscopic procedure that placed seven elastic bands on the variceal columns, with intravenous administration of terlipressin.^7^

Tests performed at this time led to a diagnosis of HCV infection, which had not been revealed previously. Hence, the patient was coinfected with HBV, HCV and HDV. 

Following the decompensation of the liver, the doctors decided not to resect the smaller nodules, and they waited to perform an OLT.

### Orthotopic liver transplantation

In June 2010, the patient's corrected MELD score was 29 and he underwent an OLT. This was followed by immunosuppressive therapy that comprised mycophenolate mofetil (720 milligrams once a day), tacrolimus (5.0 milligrams twice a day) and prednisone (20 milligrams once a day). 

During the anhepatic phase of surgery, hepatitis B immunoglobulin (HBIG) prophylaxis was not administered because entecavir therapy was maintained, the HBV levels in the serum were undetectable and the liver donor was serologically negative for total hepatitis B core antibodies. 

After transplantation, the patient's clinical status improved. The serum liver enzyme levels normalized, an RT-PCR demonstrated that HBV and HDV had been cleared out and a serological test demonstrated that seroreversion of hepatitis B surface antigen (HBsAg) had taken place. However, hepatitis B surface antigen antibodies (anti-HBs), which are the specific marker of immunity to hepatitis B virus infection, were not detected. 

An RT-PCR test showed high levels of HCV RNA genotype 1 (log 7.07). Furthermore, progressive elevation of serum ALT levels began, which reached 400 IU/l six weeks after transplantation.

### Treatment of hepatitis C recurrence

In January 2011, the patient began hepatitis C treatment, which comprised peginterferon alfa-2A (180 micrograms once a week) and ribavirin (500 milligrams twice a day), in accordance with the Ministry of Health's protocol.^8^

The patient responded well to treatment over the next few weeks, despite the presence of HCV genotype 1, which meant that he might have been less responsive to treatment. A significant decline in the HCV viral load occurred. The viral levels were undetectable after the fourth peginterferon alfa-2A dose and normal serum hepatic transaminase levels were restored.^9^

After 52 weeks of treatment and 20 months after the OLT, the patient stopped receiving hepatitis C antiviral treatment.

### Outcome

Currently, the patient is clinically stable. HBV, HCV and HDV are at undetectable levels, the serological markers HBsAg and anti-HBs are negative and the liver enzymes are within the normal ranges. Thus, he experienced triple hepatitis virus infection and achieved a sustained virological response, such that the HBV and HDV levels have been undetectable for 36 months and the HCV levels have been below the detection limit for 27 months. An ultrasound scan performed 30 months after OLT showed no signs of liver fibrosis or new nodule development.

On the other hand, the patient developed diabetes mellitus and requires insulin therapy. The patient continues to receive an immunosuppressive regimen comprising tacrolimus and mycophenolate mofetil, and he receives entecavir to prevent HBV recurrence. 

## DISCUSSION

### Pathogenesis of triple hepatitis infection

We have presented a rare case of HBV, HCV and HDV coinfection accompanied by development of cirrhosis and HCC.

The interactions among HBV, HCV and HDV are not fully understood. Studies have shown that one virus may predominate and suppress the other viruses. During coinfection, HDV negatively affects HBV and HCV replication.^10^^-^ 14

It is not clear whether our patient was coinfected with HBV, HCV and HDV before he underwent segmental hepatectomy. Initially, HBV and HDV might have predominated over HCV and suppressed it, thus leading to a false-negative serological result for HCV. Treatment with entecavir would have suppressed HBV and HDV, thereby reactivating HCV. 

**Figure 1.**
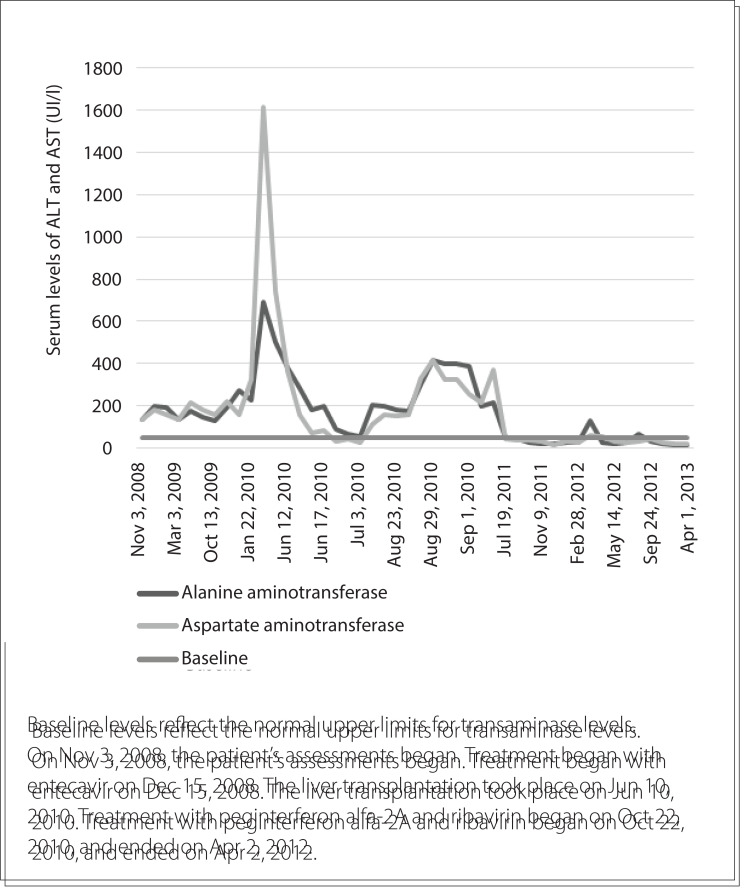
Chronological evolution of serum liver transaminase levels.

Figure 1 shows the patient's serum liver transaminase levels from the first consultation to the last consultation. 

### Prevalence of triple hepatitis infection: global and local scenarios

Triple hepatitis infection is one of the less studied coinfections. The dearth of investigations into this coinfection may be associated with its low prevalence and the high endemicity of HBV, HCV and HDV within well-demarcated areas, including the Asian mainland, eastern Europe and communities within the Amazon region of northern Brazil.^15^^-^ 19

Seroprevalence studies on triple hepatitis virus infections usually involve blood donors or patients with chronic liver diseases, but these do not represent the whole population.^15^^-^ 19 In the state of Acre in Brazil, the seroprevalence rates for HBV and HDV are approximately 3.4% and 1.7%, respectively.^20^ The biomolecular prevalence of HCV RNA within the population is estimated as 2.5%, and 0.05% of the same population will express concomitant markers, namely HCV RNA, HBsAg and antibodies against HDV.^17^

### Complications associated with coinfection: cirrhosis and hepatocellular carcinoma

Few data describing the relationship between HCC and triple infection with HBV, HCV and HDV are available. A study in Mongolia determined that triple infection with HBV, HCV and HDV was significantly more frequent among patients with HCC than among those with chronic hepatitis caused by other etiologies (P < 0.0001), and it was more common among patients with liver cirrhosis than among those with liver cirrhosis caused by other etiologies (P < 0.0001).^18^

### Liver transplantation and viral recurrence

Some authors have considered that low-dose intramuscular HBIG administered with a potent nucleos(t)ide analogue is a better prophylactic strategy for preventing graft infection in patients coinfected with HBV and HDV.^3^^,^ 24 However, the introduction of new nucleos(t)ide analogues, including entecavir and tenofovir, in the 2000s, modified the evolution of chronic liver disease caused by HBV.^25^^,^ 26

A meta-analysis reported that among HBV-negative patients, HBV recurrence rates after OLT did not differ significantly between the group that received prophylaxis following OLT using HBIG + lamivudine and the group that received prophylaxis with entecavir or tenofovir as monotherapy (P = 0.17, risk difference: 0.06, 95% confidence interval [CI]: -0.02 to 0.14). Therefore, HBIG may be unnecessary for previously HBV-negative patients.^27^ The protocols used by the Hepatology and Tropical Diseases Unit of Acre and the Liver Transplantation Group do not include intramuscular HBIG administration. 

In 2009, only 2% of liver transplantations were undertaken in patients coinfected with HBV and HDV. HBV infection recurrence in patients with liver transplants who were coinfected with HDV is less frequent than in patients infected with HBV alone (17% and 56%, respectively), which probably relates to lower HBV replication levels at the time of transplantation because of HDV coinfection.^3^

Chronic HCV infection is the leading indication for liver transplantation in Europe, South and North America, Australia and Japan.^1^^,^ 2

Most patients who have detectable levels of HCV in their serum samples and who have undergone transplantation surgery experience graft infection. This is the most frequent complication of this kind of procedure, and is associated with a faster process of fibrosis of the hepatic parenchyma.^8^

After transplantation, treatment of recurrent HCV infection remains a challenge. The development of fibrosis is accelerated following transplantation in these patients, and 20-30% of the cases progress to cirrhosis within five years of transplantation. Liver transplant recipients who have had HCV infections have lower survival rates (hazard ratio [HR]: 1.23; 95% CI: 1.12 to 1.35) and higher rates of graft failure (HR: 1.30; 95% CI: 1.21 to 1.39) than do liver transplant recipients who have not had HCV infections.^28^

In the current case, the liver was not biopsied after OLT. However, liver fibrosis and new nodules were not evident during an ultrasound scan performed 30 months after OLT.

Only 30% of the liver transplant recipients who experience HCV infection recurrences show sustained virological responses to antiviral regimens that include peginterferon alfa-2A and ribavirin,^1^ which were administered in the current case and comprised the standard treatment recommended for HCV infection in Brazil at that time.^8^

Recurrence of HCV infection is infrequent in patients who are coinfected with three different hepatitis viruses.^11^ Taniguchi et al. studied 13 patients who underwent OLT, and had HBV and HCV coinfections with and without HDV infections. Those who were not infected with HDV developed HCV recurrence after OLT, while those who were infected by HDV were negative for HCV after OLT.^29^ All of the patients received intramuscular HBIG prophylaxis, which prevented disease recurrence effectively. Two patients rapidly developed recurrences of HBV infection after HBIG was discontinued, and one patient developed HBV and HDV-related cirrhosis 30 months after OLT. There was no HCC recurrence after a median follow-up period of 38 months.^29^

We reviewed the literature in Medline, PubMed, Embase and Lilacs using the English keywords "Hepatitis B", "Hepatitis C", "Hepatitis D" and "Liver Transplantation"; the Portuguese words "Hepatite B", "Hepatite C", "Hepatite D" and "Transplante de Fígado"; and the Spanish keywords "Hepatitis B", "Hepatitis C", "Hepatitis D" and "Transplante de Hígado". We found one case report that described a patient with HCC who had been coinfected with HBV, HCV and HDV, and who underwent OLT (Table 1). The patient's cirrhosis recurred four years after OLT and retransplantation was required. However, the case report did not describe the clinical outcomes associated with the triple infection.^30^

**Table 1.** Literature search in medical databases for case reports on triple infection with hepatitis B, C and D viruses associated with hepatocellular carcinoma and treated with orthotopic liver transplantation. The literature search was conducted on July 5, 2014

Database	Search strategies	Papers found	Related papers
Medline (via PubMed)	((((Hepatitis B) AND Hepatitis C) AND Hepatitis D) AND Liver Transplantation) AND “case reports” [Publication Type]	2	1
Embase (via Elsevier)	((((Hepatitis B) AND Hepatitis C) AND Hepatitis D) AND Liver Transplantation) AND “case reports” [Publication Type]	12	0
Lilacs (via Bireme)	((Hepatite B [DeCs]) OR (Hepatitis B [MeSH])) AND ((Hepatite C [DeCs]) OR (Hepatitis C [MeSH])) AND ((Hepatite D [DeCs]) OR (Hepatitis D [MeSH])) AND ((Transplante de Fígado [DeCs]) OR (Liver Transplantation [MeSH]) OR (Trasplante de Hígado [DeCs]))	0	0

## CONCLUSIONS

In this case, antiviral therapy using entecavir administered before and after OLT was effective for sustained clearance of HBV and HDV. HBIG was not administered and the serological markers of immunity were not evident.

Recurrence of HCV infection was expected after OLT, and it was successfully treated with peginterferon alfa-2A and ribavirin.
